# Endoscopic features of gastric neuroendocrine tumors

**DOI:** 10.1002/deo2.70088

**Published:** 2025-02-26

**Authors:** Katsunori Matsueda, Noriya Uedo, Masanori Kitamura, Takashi Kanesaka, Muneshin Morita, Satoki Shichijo, Akira Maekawa, Yoji Takeuchi, Koji Higashino, Tomoki Michida, Ryu Ishihara, Seiji Kawano, Motoyuki Otsuka

**Affiliations:** ^1^ Department of Gastrointestinal Oncology Osaka International Cancer Institute Osaka Japan; ^2^ Department of Gastroenterology and Hepatology Okayama University Hospital Okayama Japan; ^3^ Department of Diagnostic Pathology and Cytology Osaka International Cancer Institute Osaka Japan; ^4^ Department of Gastroenterology and Hepatology Gunma University Graduate School of Medicine Gunma Japan

**Keywords:** endoscopic feature, magnifying narrow‐band imaging, neuroendocrine tumor, stomach, white‐light imaging

## Abstract

**Objectives:**

The endoscopic features of gastric neuroendocrine tumors (G‐NETs) remain unclarified. The present study investigated the endoscopic features of G‐NETs in relation to the clinicopathological findings.

**Methods:**

This retrospective study analyzed consecutive patients with G‐NETs who received endoscopic or surgical treatment between January 2005 and December 2023. The endoscopic and clinicopathological findings of the lesions were analyzed to provide diagnostic information.

**Results:**

Among 29 patients, the characteristic endoscopic findings of G‐NETs on white‐light images were reddish color (66%), dilated vessels (83%), submucosal tumor‐like marginal elevation (59%), and central depression (48%). The gross appearance of G‐NETs was classified into two macroscopic subtypes: reddish polypoid lesions (*n* = 17) and submucosal tumor‐like lesions (*n* = 9). Magnifying narrow‐band imaging endoscopy revealed an absent microsurface pattern plus an irregular microvascular pattern in all cases of reddish polypoid lesions with central depressions (100%, 9/9). The findings of a reddish polypoid lesion and an absent microsurface pattern plus an irregular microvascular pattern corresponded to the subepithelial NET component close to the non‐neoplastic surface epithelium. Additionally, reddish polypoid lesions were significantly more frequent in type 1 G‐NETs than in type 3 G‐NETs (80% vs. 11%, *p *< 0.001), while submucosal tumor‐like lesions were significantly more frequent in type 3 G‐NETs than in type 1 G‐NETs (78% vs. 10%, *p *< 0.001).

**Conclusions:**

These endoscopic features should increase the index of suspicion and help clinicians to correctly diagnose G‐NETs through the pathological examination of biopsy specimens.

## INTRODUCTION

The 2019 World Health Organization (WHO) classification defined epithelial neoplasms with predominant neuroendocrine differentiation as neuroendocrine neoplasms (NENs)^1^. NENs are classified into well‐differentiated neuroendocrine tumors (NETs) and poorly differentiated neuroendocrine carcinomas (NECs) based on the degree of histological differentiation. NETs are further classified as grade 1–3 (NET‐G1, NET‐G2, and NET‐G3) based on their mitotic count and cell proliferation index.

Rindi et al.^2^, ^3^ divided gastric NETs (G‐NETs) into three distinct subtypes with different clinical backgrounds: type 1 G‐NETs (approximate prevalence 75%–80%) associated with chronic atrophic gastritis (i.e., autoimmune gastritis); type 2 G‐NETs (prevalence 5%) associated with multiple endocrine neoplasia Zollinger‐Ellison syndrome; and type 3 G‐NETs (prevalence 15%–25%) that are sporadic lesions without specific background diseases. Types 1 and 2 G‐NETs are associated with hypergastrinemia and hyperplasia of enterochromaffin‐like (ECL) cells, while type 3 G‐NETs grow without hypergastrinemia. The prognoses of types 1 and 2 G‐NETs are typically excellent, whereas type 3 G‐NETs are aggressive and frequently present with lymphovascular invasion and lymph node metastasis. Thus, the clinical classification affects the selected treatment strategies, such as endoscopic surveillance, endoscopic resection, or gastrectomy with lymphadenectomy. Although these clinicopathological features have been extensively studied,^2^, ^4^, ^5^, ^6^, ^7^ information on the endoscopic features of G‐NETs, particularly the magnifying narrow‐band imaging (M‐NBI) endoscopic features, is limited. We therefore investigated the endoscopic and clinicopathological findings to elucidate the endoscopic characteristics of G‐NETs.

## METHODS

### Study design and setting

This retrospective study was conducted at a Japanese referral cancer center. The study protocol was approved by the Institutional Review Board on February 14, 2024 (22166‐2), and the study complied with the Helsinki Declaration of 1964 and later versions. The need for informed consent for study participation was waived because this was a retrospective study of anonymized data.

### Participants

Consecutive patients with gastric tumors, including a NET component based on the histological diagnosis of an endoscopic biopsy or resected specimens from January 2005 to December 2023, were identified in our pathology database. Among these, patients with G‐NETs were eligible for inclusion in this study. Patients with NET‐G3 were excluded because a NET‐G3 was classified as an NEC in the WHO 2010 classification^8^. Patients with G‐NETs not treated by endoscopic or surgical resection were also excluded because of reported inconsistencies in the tumor grade between biopsy and final resected histology findings^9^.

### Data collection

The collected data included age, sex, endoscopic findings on white‐light imaging (WLI) and M‐NBI, gastric mucosal atrophy, presence of anti‐parietal cell antibodies (APC‐Ab), *Helicobacter pylori* infection, serum gastrin concentration, treatment method, and histological findings such as tumor size, invasion depth, tumor grade, and lymphovascular invasion. Gastric mucosal atrophy was classified into C‐1, C‐2, C‐3, O‐1, O‐2, and O‐3 in accordance with Kimura‐Takemoto classification^10^, with C‐1 defined as negative and the remainder defined as positive. *H. pylori* infection was assessed by serum antibody against *H. pylori* or histological examination. A positive result in either test indicated *H. pylori* infection. In patients with multiple G‐NETs, only the lesion with the most advanced histology or the largest lesion was studied.

### Assessment of endoscopic findings

**FIGURE 1:**
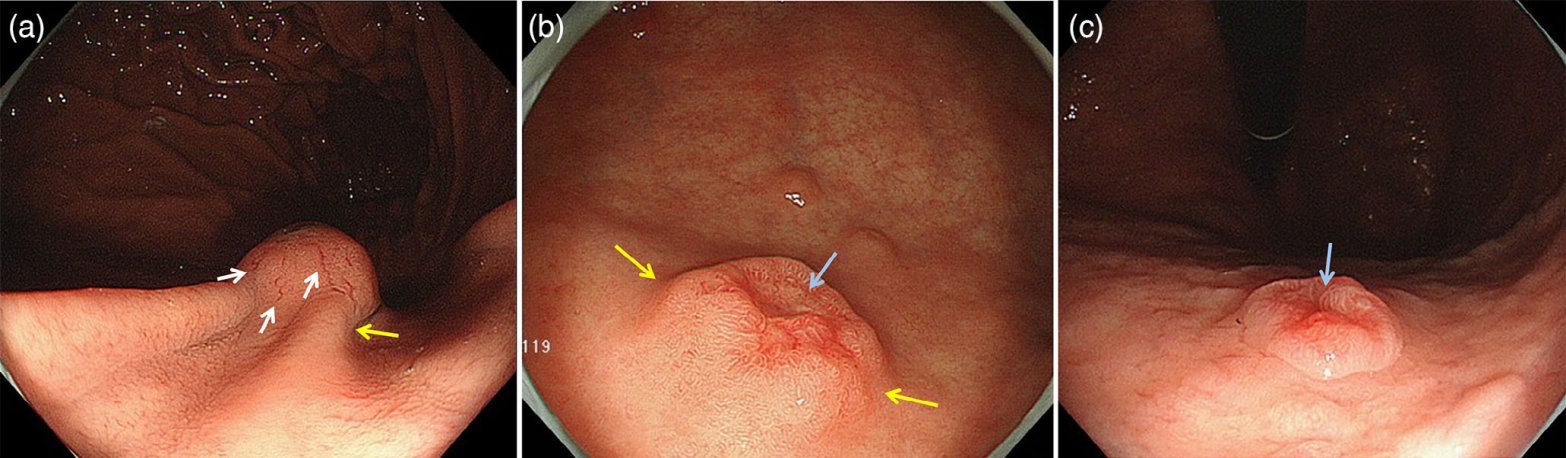
Distinctive endoscopic features of gastric neuroendocrine tumors: (a) dilated vessels (white arrows) and submucosal tumor‐like marginal elevation (yellow arrow), (b) submucosal tumor‐like marginal elevation (yellow arrows) and a central depression (light blue arrow), and (c) central depression (light blue arrow).

**FIGURE 2:**
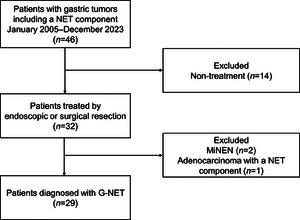
Flowchart of patient selection: NET, neuroendocrine tumor; G‐NET, gastric neuroendocrine tumor; MiNEN, mixed neuroendocrine‐non‐neuroendocrine neoplasm.

**FIGURE 3:**
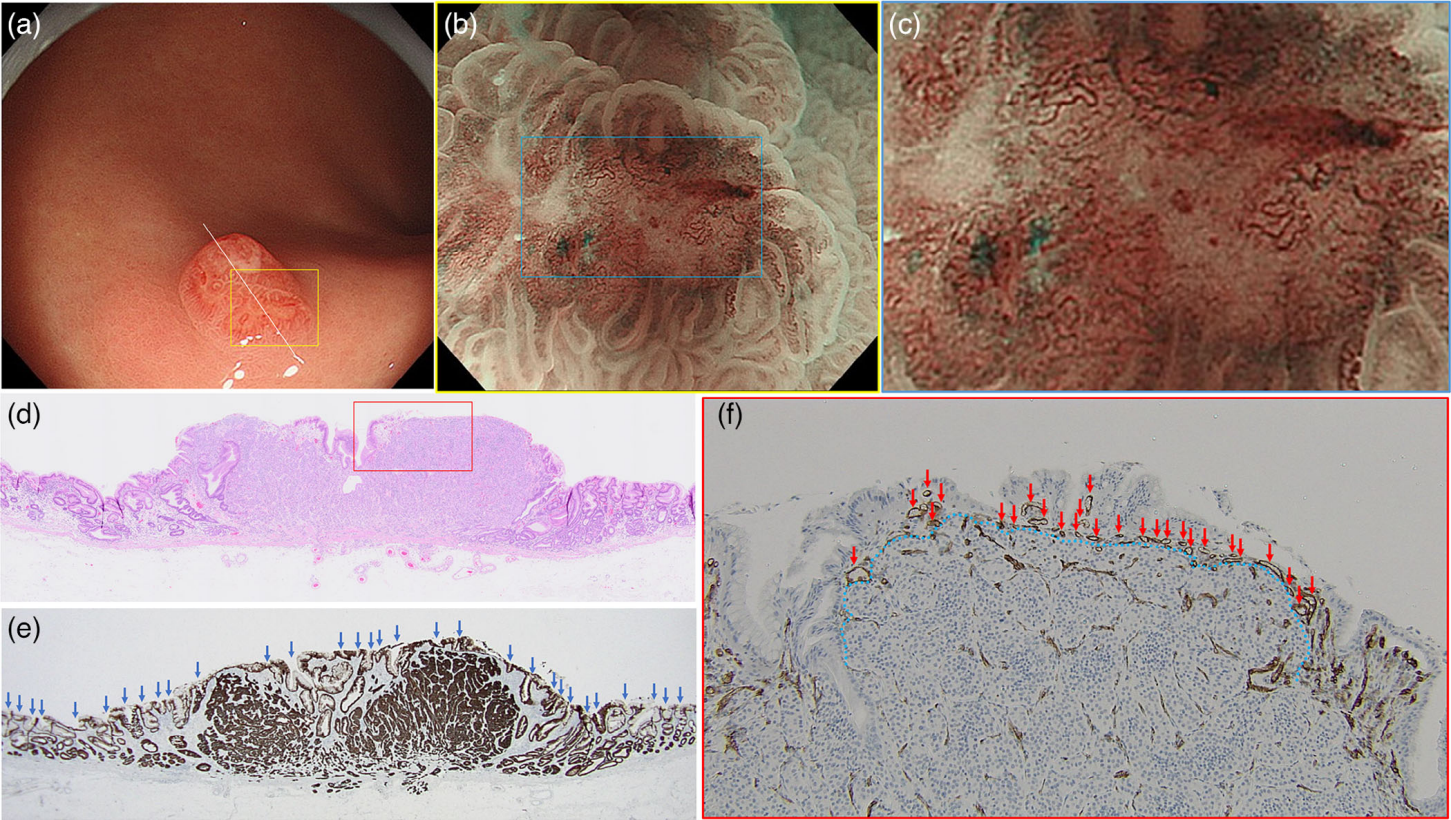
Representative case of the reddish polypoid lesion: (a) white‐light imaging shows a reddish superficial elevated lesion with a central depression on the posterior wall in the middle third of the stomach, (b) magnifying narrow‐band imaging reveals an absent microsurface pattern plus an irregular microvascular pattern in the depressed area, which is surrounded by an enlarged groove‐type mucosal structure (yellow box in Figure 3a), (c) enlarged magnifying narrow‐band imaging of the depressed area in the blue box in Figure 3b. The brownish irregular microvessels consist of closed (connected) looped vessels, (d) Histological examination of the endoscopic submucosal dissection specimen with hematoxylin and eosin staining along the cut surface of the white line in Figure 3a. The neuroendocrine tumor (NET) is composed of small uniform cells in nests predominantly located in the mucosa just below the thinned surface epithelium (magnification ×20), (e) immunohistochemical examination for cytokeratin AE1/AE3 confirms that the non‐neoplastic surface epithelium remains thin above the NET. The presence of intramucosal NET component causes non‐neoplastic gland ducts to become sparse and shallow, resulting in the extended intervening parts between the gastric pits (indicated by blue arrows) compared with the surrounding mucosa (magnification ×20), and (f) immunohistochemical examination for CD31 in the area in the red box in Figure 3d reveals dense dilated subepithelial capillaries (red arrows) in close contact on the tumor surface (light blue dashed line), flattening the surface epithelium and thinning the superficial layer of the lamina propria (magnification ×100). These findings indicate that the NET component quite close to the surface epithelium histologically corresponds to an absent microsurface pattern plus an irregular microvascular pattern on magnifying narrow‐band imaging.

Endoscopic findings were retrieved from the endoscopy report and confirmed by the investigators (Katsunori Matsueda and Noriya Uedo) after reviewing the endoscopic images stored on an endoscopic image server (Solemio ENDO version 3.3; Olympus Medical Systems). Prior to 2005, examinations were carried out with WLI using non‐magnifying endoscopes (EVIS GIF‐XQ‐240 or ‐Q240; Olympus), and then magnifying endoscopes (EVIS GIF‐Q240Z, ‐H260Z, ‐H290Z, or ‐XZ1200; Olympus) were used with NBI in most cases. The macroscopic type was assessed in accordance with the Japanese classification of gastric carcinoma^11^. The lesion color was classified as reddish, isochromatic, or whitish in comparison with the surrounding mucosa. On WLI, the prevalence of the known three characteristic features of G‐NETs was evaluated: (1) dilated vessels (Figure 1a), (2) submucosal tumor (SMT)‐like marginal elevation (Figure 1a,b), and (3) central depression (Figure 1b,c).^12^, ^13^, ^14^, ^15^, ^16^ SMT‐like marginal elevation was defined as a finding in which the margins of the elevation were covered by non‐neoplastic surrounding mucosa. For M‐NBI endoscopic findings, the microsurface (MS) and microvascular (MV) patterns were classified as regular, irregular, or absent^17^.

Two endoscopists (Katsunori Matsueda and Noriya Uedo) reviewed all recorded endoscopic images, diagnosed the endoscopic findings, and rechecked the endoscopic images if the diagnoses differed.

### Evaluation of histological findings

All G‐NETs were diagnosed histologically in accordance with the WHO 2019 classification^18^. Well‐differentiated NETs with a Ki‐67 index of ≤20% were defined as NETs (G1/G2). On the basis of the WHO classification, epithelial tumors containing a mixture of NEN and non‐NEN with ≥30% of each component in the tumor were classified as mixed neuroendocrine‐non‐NENs (MiNENs). G‐NET was diagnosed based on a NET (G1/G2) component of ≥70%.

The degree of histological differentiation, tumor size, invasion depth, tumor grade, and lymphovascular invasion was evaluated by an expert gastrointestinal pathologist (Masanori Kitamura) who was blinded to the clinical information. All G‐NET cases were reconfirmed as having neuroendocrine differentiation by immunohistochemical staining for chromogranin A, synaptophysin, and CD56. The tumors were classified by Ki‐67 proliferation indices as G1 (<3%) or G2 (3%–20%). The presence of ECL cell hyperplasia and endocrine cell micronests was assessed in the surrounding non‐neoplastic mucosa.

### Clinical classification of G‐NETs

The diagnosis of type 1 G‐NET was based on the following criteria: (i) no evidence of multiple endocrine neoplasia Zollinger‐Ellison syndrome, (ii) APC‐Ab positivity, or (iii) presence of ECL cell hyperplasia/endocrine cell micronests and fundic gland atrophy in histology; or (iv) evidence of hypergastrinemia (>450 pg/mL) in cases with no evidence of APC‐Abs or ECL cell hyperplasia/endocrine cell micronests. Type 2 G‐NETs were defined as those with the presence of multiple endocrine neoplasia type I or Zollinger‐Ellison syndrome. Tumors that did not meet the criteria for types 1 and 2 were classified as type 3 G‐NETs.

### Treatment strategy

The initial treatment method of G‐NETs, endoscopic or surgical resection, was determined based on a policy following the Clinical Practice Guidelines for Gastroenteropancreatic Neuroendocrine Neoplasms in Japan^19^. For type 1 G‐NETs, endoscopic resection was proposed for tumors ≤1 cm, with no more than several tumors confined to the submucosa without lymph node metastasis. On the other hand, surgical resection was essentially indicated for type 3 G‐NETs and tumors that did not meet the criteria for endoscopic resection.

### Measured outcomes

The primary analysis aimed to clarify the prevalence of characteristic endoscopic findings of G‐NETs. To elucidate the histological findings behind the characteristic endoscopic findings of the G‐NET, the predominance of tumor localization in the mucosa or submucosa and the distance between the tumor and the surface epithelium were assessed. The tumor localization was determined as mucosa‐predominant or submucosa‐predominant depending on whether the tumor epicenter was above or below the muscularis mucosae in the specimen with the maximum tumor diameter. The closest distance between the tumor edge and the surface epithelium was measured using a virtual pathology system (cellSens V3.1 imaging software; Olympus Corp.).

Ancillary analyses compared the endoscopic findings of G‐NETs in accordance with the clinical classification.

### Statistical analyses

Continuous variables were reported as median (range) and compared using Wilcoxon rank sum test, while categorical variables were reported as frequency (percentage) and compared using Pearson's χ^2^ test or Fisher's exact test. *p *< 0.05 was considered statistically significant. All statistical analyses were performed using JMP 17 software (SAS Institute Inc.).

## RESULTS

### Patient and tumor characteristics

Forty‐six patients with gastric tumors with a NET component were identified during the study period. Among these, 14 patients who did not undergo resection, two with MiNENs, and one with adenocarcinoma with a NET component of <30% were excluded, leaving 29 patients with G‐NETs for analysis (Figure 2).

The clinicopathological data of the 29 patients and their main lesions are shown in Table 1. Eleven patients (38%) had multiple lesions. The main initial treatment methods were endoscopic treatment, endoscopic mucosal resection (*n* = 11, 38%), and endoscopic submucosal dissection (*n* = 11, 38%). The median histological tumor size was 5 (range: 1–35) mm. There were 25 G1 lesions (86%) and four G2 lesions (14%).

**TABLE 1:** Clinicopathological characteristics of 29 patients with gastric neuroendocrine tumors.

Clinicopathological findings	Total (*n* = 29)
Age in years, median (range)	64 (33–82)
Sex, *n* (%)	
Male	14 (48)
Female	15 (52)
Number of tumors, *n* (%)	
Single	18 (62)
Multiple	11 (38)
Initial treatment, *n* (%)	
EMR	11 (38)
ESD	11 (38)
Surgery	7 (24)
Histological tumor size in mm, median (range)	5 (1–35)
Histological depth, *n* (%)	
T1a (M)	10 (34)
T1b (SM)	19 (66)
Tumor grade, *n* (%)	
Grade 1	25 (86)
Grade 2	4 (14)
Lymphovascular involvement, *n* (%)	
Present	4 (14)
Absent	25 (86)
APC‐Ab, *n* (%)	
Positive	1 (3)
Negative	2 (7)
Not tested	26 (90)
*H. pylori* infection	
Positive	0
Negative	24 (83)
Not tested	5 (17)
Serum gastrin concentration in pg/mL, median (range)	721 (61–9760)
Hypergastrinemia	
Present	10 (34)
Absent	8 (28)
Not tested	11 (38)
ECL cell hyperplasia or ECM in the background mucosa, n (%)	
Present	18 (62)
Absent	10 (34)
Not tested	1 (3)
Clinical classification by Rindi et al., *n* (%)	
Type 1	20 (69)
Type 3	9 (31)

Abbreviations: APC‐Ab, anti‐parietal cell antibodies; ECL, enterochromaffin‐like; ECM, endocrine cell micronest; EMR, endoscopic mucosal resection; ESD, endoscopic submucosal dissection; M, mucosa; SM, submucosa.

### Characteristic endoscopic findings

The endoscopic findings of the 29 G‐NETs are summarized in Table 2. M‐NBI was evaluable in 21 cases.

**TABLE 2:** Endoscopic characteristics of gastric neuroendocrine tumors.

Endoscopic findings	Total (*n* = 29)	Clinical classification by Rindi et al.		
		Type 1 (*n* = 20)	Type 3 (*n* = 9)	*p‐*value
Gastric mucosal atrophy, *n* (%)				
Absent	7 (24)	0	7 (78)	<0.001
Present	22 (76)	20 (100)	2 (22)	
Gastric mucosal atrophy by Kimura‐Takemoto classification, *n* (%)				<0.001
C‐1	7 (24)	0	7 (78)	
C‐2	0	0	0	
C‐3	0	0	0	
O‐1	0	0	0	
O‐2	6 (21)	5 (25)	1 (11)	
O‐3	16 (55)	15 (75)	1 (11)	
Histological tumor size in mm, median (range)	5 (1–35)	6 (2–27)	5 (1–35)	0.685
Location in the stomach, *n* (%)				
Upper third	14 (48)	9 (45)	5 (56)	0.599
Middle third	15 (52)	11 (55)	4 (44)	
Tumor color, *n* (%)				
Reddish	19 (66)	18 (90)	1 (11)	<0.001
Isochromatic	10 (34)	2 (10)	8 (89)	
Macroscopic type, *n* (%)				
Advanced cancer‐like protruding lesion	2 (7)	1 (5)	1 (11)	0.548
Superficial protruded/elevated lesion	24 (83)	17 (85)	7 (78)	0.634
Superficial depressed lesion	3 (10)	2 (10)	1 (11)	0.928
Dilated vessels, *n* (%)				
Present	24 (83)	18 (90)	6 (67)	0.124
Absent	5 (17)	2 (10)	3 (33)	
SMT‐like marginal elevation, *n* (%)				
Present	17 (59)	10 (50)	7 (78)	0.160
Absent	12 (41)	10 (50)	2 (22)	
Central depression, *n* (%)				
Present	14 (48)	14 (70)	0	<0.001
Absent	15 (52)	6 (30)	9 (100)	
Macroscopic subtype, *n* (%)				
Reddish polypoid lesion	17 (59)	16 (80)	1 (11)	<0.001
SMT‐like lesion	9 (31)	2 (10)	7 (78)	<0.001
Unclassifiable	3 (10)	2 (10)	1 (11)	0.928
MS pattern on M‐NBI^*^, *n* (%)				
Regular	11 (52)	3 (23)	8 (100)	<0.001
Irregular	1 (5)	1 (8)	0	0.422
Absent	9 (43)	9 (69)	0	0.002
MV pattern on M‐NBI^*^, *n* (%)				
Regular	10 (48)	3 (23)	7 (88)	0.004
Irregular	11 (52)	10 (77)	1 (13)	0.004
Absent	0	0	0	–
Absent MS pattern plus irregular MV pattern^*^, *n* (%)				
Present	9 (43)	9 (69)	0	0.002
Absent	12 (57)	4 (31)	8 (100)	

Abbreviations: M‐NBI, magnifying narrow‐band imaging; MS, microsurface; MV, microvascular; SMT, submucosal tumor.

* M‐NBI findings were not available for seven cases and were not evaluable because of an adherent white coat in one case.

Nineteen lesions (66%) were reddish in color. Twenty‐six lesions (90%) presented as superficial elevated (0‐IIa) or protruded (0‐I or 1) lesions. The characteristic features of G‐NETs on WLI were frequently prevalent: dilated vessels (*n* = 24, 83%), SMT‐like marginal elevation (*n* = 17, 59%), and a central depression (*n* = 14, 48%).

**FIGURE 4:**
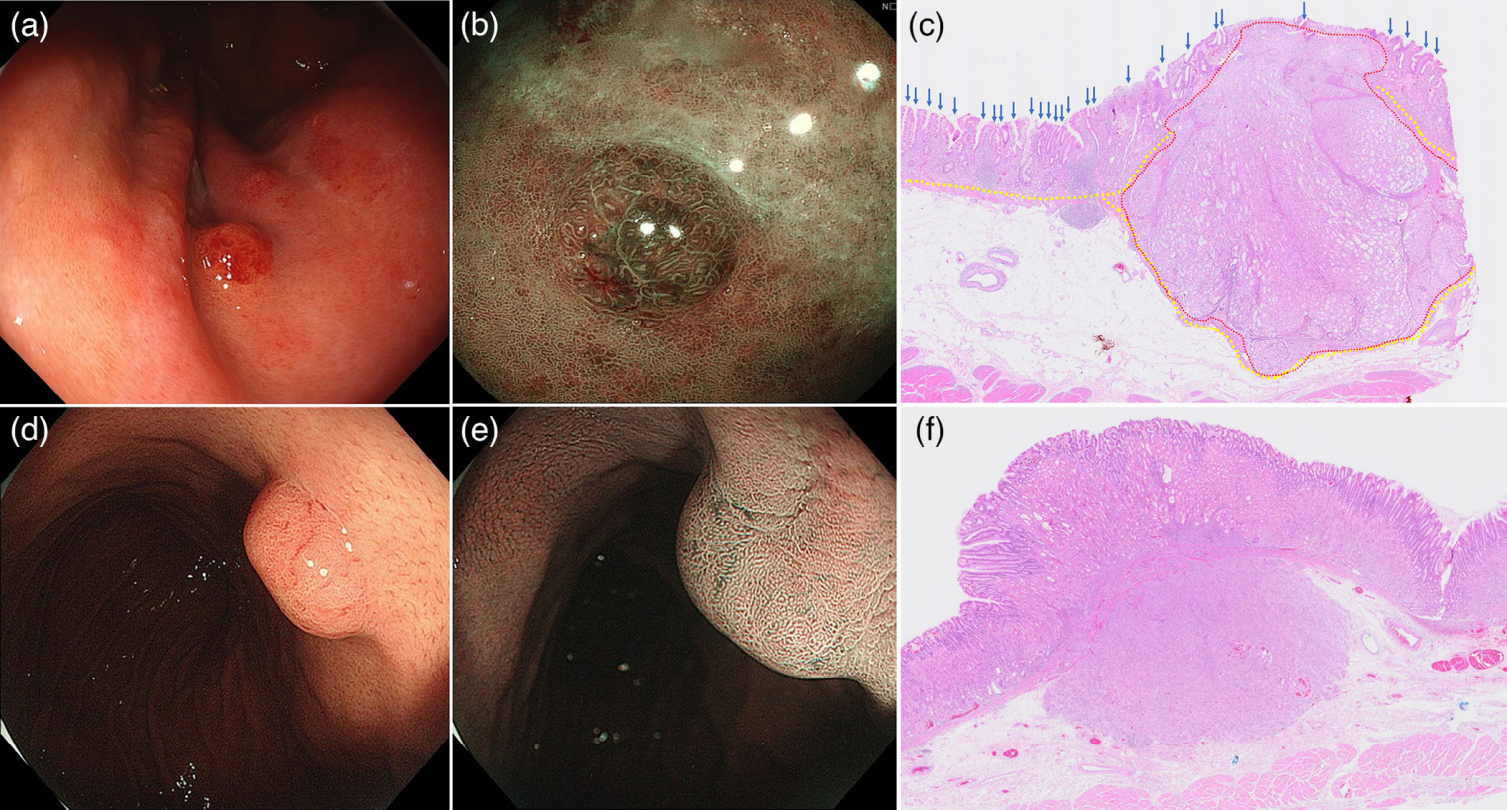
Representative cases of type 1 and type 3 gastric neuroendocrine tumors (G‐NETs) Type 1 G‐NET: (a) white‐light imaging shows a reddish superficial protruded lesion (i.e., reddish polypoid lesion) on the greater curvature in the upper third of the stomach, (b) magnifying narrow‐band imaging shows a regular microsurface pattern plus a regular microvascular pattern on an enlarged groove‐type mucosa, (c) histological examination of the surgical specimen with hematoxylin and eosin staining reveals that the intramucosal neuroendocrine tumor (NET) close to the epithelial surface makes the gastric gland ducts sparse and shallow, resulting in the extended intervening parts between the gastric pits (blue arrows). The tumor border and muscularis mucosa are indicated by red and yellow dashed lines, respectively (magnification ×20) Type 3 G‐NET, (d) White‐light imaging shows isochromatic submucosal tumor‐like lesions on the lesser curvature in the upper third of the stomach, (e) magnifying narrow‐band imaging reveals a regular microsurface pattern plus a regular microvascular without a demarcation line, and (f) histological examination of the surgical specimen with hematoxylin and eosin staining reveals that the NET is predominantly located in the submucosa beneath the non‐atrophic mucosa, where the normal gland ducts remain intact (magnification ×20).

The G‐NETs were classified into two macroscopic subtypes. (1) Reddish polypoid lesions (*n* = 17, 59%; Figures 1b,c, 3a, and 4a) with an enlarged groove‐type mucosal structure. A central depression was seen in 14 lesions (14/17, 82%). All lesions in which MS and MV patterns were evaluable in the central depression under M‐NBI had an absent MS pattern plus an irregular MV pattern (9/9, 100%, Figure 3b,c). (2) SMT‐like lesions (*n* = 9, 31%; Figures 1a and 4d). Eight of these were isochromatic (89%). All lesions in which MS and MV patterns were evaluable showed a regular MS plus a regular MV pattern without a demarcation line (100%, 7/7; Figure 4e). The other three cases (10%) were unclassifiable.

In 27 cases, histological slides were available for detailed reassessment of histological findings (Table 3). Fifteen G‐NETs (56%) were predominantly located in the mucosa. The reddish polypoid lesions had a significantly shorter distance between the tumor and the surface epithelium than other lesions (*p *= 0.001), irrespective of the predominance of tumor localization in the mucosa or submucosa (*p *= 0.381, Figures 3 and 4). The finding of an absent MS pattern plus an irregular MV pattern on M‐NBI was also associated with a significantly shorter distance between the tumor and the surface epithelium than in lesions without this finding (*p *= 0.006), irrespective of the predominance of tumor localization (*p *= 0.801, Figure 3).

**TABLE 3:** Histological findings associated with characteristic endoscopic findings.

		**Endoscopic findings of G‐NET**					
**Histological findings**	**Total** **(*n* = 27)** ^*^	**Reddish polypoid lesion** **(*n* = 16)**	**Others** **(*n* = 11)**	***p‐*value**	**Absent MS+irregular MV present** **(*n* = 9)** ^**^	**Absent MS+irregular MV absent** **(*n* = 12)** ^**^	***p‐*value**
Tumor localization, *n* (%)				0.381			0.801
Mucosa predominant	15 (56)	10	5		5	6	
Submucosa predominant	12 (44)	6	6		4	6	
Distance from the epithelium in µm, median (range)	76 (0–2239)	36 (0–288)	387 (0–2239)	0.001	36 (0–288)	290 (26–2239)	0.006

Abbreviations: G‐NET, gastric neuroendocrine tumor; MS, microsurface; MV, microvascular.

* The histological slides were not evaluated for two cases because the specimens had been discarded.

** Magnifying narrow‐band imaging findings were available for 21 cases.

### Endoscopic findings in accordance with the clinical classification

There were no type 2 G‐NETs. APC‐Ab testing was performed in three cases, among which one was positive. Therefore, the clinical subtypes were classified mainly based on the histological findings of the surrounding mucosa or evidence of hypergastrinemia (Table 1). Accordingly, 20 lesions (69%) were diagnosed as type 1 G‐NETs and nine (31%) were classified as type 3 G‐NETs.

Table 2 lists the endoscopic findings of G‐NETs grouped by clinical classification. Gastric mucosal atrophy was more frequently observed in type 1 than in type 3 G‐NETs (100% vs. 22%, *p *< 0.001). Regarding the endoscopic features on WLI, type 1 G‐NETs were more frequently a reddish color, while type 3 G‐NETs were more frequently isochromatic (*p *< 0.001, Figure 4). A central depression was more frequent in type 1 G‐NETs than in type 3 G‐NETs (70% vs. 0%, *p *< 0.001). Regarding the macroscopic subtypes, reddish polypoid lesions were significantly more frequent in type 1 G‐NETs than in type 3 G‐NETs (80% vs. 11%, *p *< 0.001), whereas SMT‐like lesions were significantly more frequent in type 3 G‐NETs than in type 1 G‐NETs (78% vs. 10%, *p *< 0.001). On M‐NBI endoscopy findings, an absent MS pattern plus an irregular MV pattern was significantly more frequent in type 1 G‐NETs than in type 3 G‐NETs (69% vs. 0%, *p *= 0.002).

## DISCUSSION

To the best of our knowledge, this is the first study to elucidate the characteristic endoscopic findings of G‐NETs in relation to histological findings.

G‐NETs originate from endocrine progenitor cells in the basal layer of the mucosa and then gradually proliferate and expand into the mucosa and submucosa, lifting up the surrounding non‐neoplastic mucosa.^4^, ^12^ This growth pattern explains the appearance of SMT‐like marginal elevation. Dilated vessels appeared to result from congestion caused by pressure from the subepithelial tumor and elevation of the vessels to the superficial layer of the mucosa. Although NETs have characteristics of SMTs, neoplastic endocrine cells proliferate within both the lamina propria and the submucosa. In our study, more than half (56%) of the lesions were predominantly located in the mucosa, thereby forming a tumor frequently accompanied by epithelial changes such as a reddish color or central depression.^12^, ^20^ The most characteristic WLI endoscopic findings of G‐NETs were a combination of SMT‐like marginal elevation, a reddish color, and a central depression, which are features that indicate subepithelial tumor growth.

The G‐NETs had two major macroscopic subtypes, of which the most common was a reddish polypoid lesion. The reddish polypoid lesions were characterized by an enlarged groove‐type mucosal structure with/without a central depression. The extended intervening part between the gastric pits in the enlarged groove‐type mucosal structure was attributed to the histological finding that gastric pits above the NET became sparse and shallow owing to the presence of the subepithelial tumor near the surface epithelium (Figures 3e and 4c). We hypothesized that the reddish polypoid appearance might be formed by intramucosal tumor proliferation toward the epithelial surface. Accordingly, although the predominant proliferation of the tumor in the mucosa was not associated with the reddish polypoid appearance, we confirmed that a shorter distance between the tumor and the surface epithelium was significantly associated with the reddish polypoid appearance of the G‐NETs.

Significantly more epithelial changes such as a reddish color, central depression, or enlarged groove‐type mucosal structure were observed in type 1 G‐NETs. In contrast, type 3 G‐NETs were a non‐reddish color without epithelial changes (i.e., SMT‐like lesions). The differences in endoscopic findings between types 1 and 3 G‐NETs may be explained by the thickness of the background gastric mucosa. Both type 1 and type 3 G‐NETs originate from the basal layer of the fundic glands; however, due to mucosal atrophy in the background gastric mucosa, type 1 G‐NETs are located closer to the surface epithelium than type 3 G‐NETs. Therefore, type 1 G‐NETs are more likely to affect the epithelium, leading to a reddish polypoid appearance that is significantly associated with the shorter distance between the tumor and the surface epithelium. In addition, as gastric glands are sparsely distributed in the atrophic background mucosa of type 1 G‐NETs, the tumor cells easily extend upwards through the non‐neoplastic gastric glands (Figure 4a–c)^21^. However, as the gastric mucosa is thick and the non‐atrophic gland ducts remain dense in type 3 G‐NETs, most of the surface epithelium is unaffected by the tumor, resulting in SMT‐like lesions (Figure 4d–f). In endoscopic screening/surveillance for G‐NETs in patients with autoimmune gastritis, it is important to pay attention to identifying reddish polypoid lesions rather than yellowish SMT‐like lesions like rectal NETs^22^.

The unique M‐NBI endoscopic finding in type 1 G‐NETs was an absent MS pattern plus an irregular MV pattern in the depressed area of reddish polypoid lesions. The MS pattern on M‐NBI is visible when epithelium with glandular structure is exposed to the surface. In G‐NETs, endocrine cells form solid nests or trabeculae structures instead of glandular structures.^23^, ^24^ Moreover, although non‐neoplastic surface epithelium was preserved in most cases, the surface epithelium above type 1 G‐NETs was flat‐thinned with few non‐neoplastic gland ducts. Thus, we consider that the absent MS pattern in type 1 G‐NETs is attributed to the histological characteristic of G‐NETs and the flattened structure of the non‐neoplastic surface epithelium. Regarding the irregular MV patterns seen in type 1 G‐NETs in our study, there was debate among the investigators as to whether these vessels were neoplastic or non‐neoplastic. Some studies have described the dilated brownish microvessels in the central depression of G‐NETs as corkscrew‐shaped.^14^, ^16^, ^25^ However, our detailed inspection of the MV pattern found that most irregular microvessels in the central depression of the G‐NETs were shaped as a connected loop (Figure 3b,c). The connected loop vessels or fine network patterns are M‐NBI endoscopic findings of a differentiated‐type adenocarcinoma forming glandular structures.^26^, ^27^ If these irregular microvessels in the depressed area are tumor vessels, they should be non‐connected loop vessels (corkscrew pattern) like in undifferentiated‐type adenocarcinomas because the NET does not form glandular structures. Furthermore, we found that the absent MS pattern plus irregular MV pattern was seen even when the tumor was up to 288 µm away from the surface epithelium. Accordingly, we speculate that the irregular MV pattern in the central depression of type 1 G‐NETs reflects a dense dilated non‐neoplastic subepithelial capillary network near the tumor surface as a result of congestion caused by an expansively growing subepithelial tumor. We believe that this M‐NBI endoscopic finding is useful to increase the index of suspicion for type 1 G‐NET and perform targeted forceps biopsy to make an accurate diagnosis.

The present study has some limitations. First, it was a retrospective, single‐center study with a small sample size. In particular, because type 2 G‐NETs were not included in the study, the endoscopic features found in this study may not be applicable to all types of G‐NETs. The limited number of patients was due to the rarity of the disease; however, the study was conducted in a specialized cancer center where all endoscopic images and histological slides were well stored. Furthermore, we carefully reviewed all the stored endoscopic and histological information to identify any associations. Our results warrant future validation in large‐scale studies of the endoscopic diagnosis of G‐NETs. Second, patients with G‐NETs who did not undergo endoscopic or surgical resection were excluded, leading to selection bias. However, we considered it important to only include patients who had undergone complete histological evaluation of the entire resected specimen to ensure an accurate diagnosis of G‐NETs (G1/G2). Third, the sensitivity of characteristic endoscopic findings for G‐NETs was assessed, but the specificity could not be determined because not all cases of non‐G‐NETs were included. However, the classification of the two macroscopic subtypes of G‐NETs defined in this study is important for clinicians to detect G‐NETs based on the presence or absence of atrophic gastritis in the background mucosa and determine a treatment strategy for G‐NETs based on the clinical classification.

In conclusion, the characteristic findings of G‐NETs on WLI were a reddish color, dilated vessels, SMT‐like marginal elevation, and a central depression. The characteristic endoscopic findings of type 1 G‐NETs were a reddish polypoid lesion with a central depression in which there was an absent MS pattern plus an irregular MV pattern on M‐NBI. Knowledge of these endoscopic features will improve the diagnostic yield of G‐NETs.

## CONFLICT OF INTEREST STATEMENT

Noriya Uedo received honoraria for his lectures from Olympus, Fujifilm, Boston Scientific (Japan), Daiichi Sankyo, Takeda Pharmaceutical, EA Pharma, Otsuka Pharmaceutical, AstraZeneca, Miyarisan Pharmaceutical, AI Medical Service, MC Medical, and Ryobi Systems. These organizations had no role in the design, practice, or analysis of this manuscript. The other authors declare no conflict of interest.

## ETHICS STATEMENT

**Approval of the research protocol by an Institutional Reviewer Board**: The study protocol was approved by the Institutional Review Board on February 14, 2024 (22166‐2).
